# Pedestrian Navigation Using Foot-Mounted Inertial Sensor and LIDAR

**DOI:** 10.3390/s16010120

**Published:** 2016-01-19

**Authors:** Duy Duong Pham, Young Soo Suh

**Affiliations:** Department of Electrical Engineering, University of Ulsan, Namgu, Ulsan 680-749, Korea; duyduongd2@gmail.com

**Keywords:** inertial sensor, IMU, distance sensor, LIDAR, pedestrian navigation, Kalman filters

## Abstract

Foot-mounted inertial sensors can be used for indoor pedestrian navigation. In this paper, to improve the accuracy of pedestrian location, we propose a method using a distance sensor (LIDAR) in addition to an inertial measurement unit (IMU). The distance sensor is a time of flight range finder with 30 m measurement range (at 33.33 Hz). Using a distance sensor, walls on corridors are automatically detected. The detected walls are used to correct the heading of the pedestrian path. Through experiments, it is shown that the accuracy of the heading is significantly improved using the proposed algorithm. Furthermore, the system is shown to work robustly in indoor environments with many doors and passing people.

## 1. Introduction

Although the global position system (GPS) is normally used in pedestrian navigation systems [1,2], alternative techniques are needed for GPS-denied environments such as indoors [3,4,5,6,7,8,9], urban canyons [10,11], underground [12,13,14], and mountain regions where GPS signals are weak or unavailable. Two research methods are mainly used in the indoor position estimation [15,16,17,18,19,20]. The first method is based on an existing network of receivers or transmitters placed at known locations. This method is known as beacon-based navigation in which position is estimated using triangulation (or trilateration) method from measured ranges (or angles). The method usually uses different technologies such as vision, ultrasound or short range radio which are generally named local positioning systems (LPSs). The survey of LPSs can be found in [15]. The second method is based on dead reckoning algorithms using sensors installed on a person or an object to locate them and known as beacon-free navigation. Since there is no environment installation requirement, this method is preferred in some applications. Several dead reckoning approaches using inertial measurement unit (IMUs) have been proposed. In [16,21], the position of a person is estimated using an inertial navigation algorithm (INA). Since its accuracy degrades over time, additional sensors are often used with IMUs such as a time of arrival based on LPSs [17], received signal strength (RSS) based on LPSs [16] or distance sensors [18,19,20].

In [16], persons are accurately located by combining an active RFID technology with an inertial navigation system in which the received signal strengths obtained from several active RFID tags are used to aid a foot-mounted IMU based position estimation. This method requires that RFID tags be installed at the known locations. In [17], the RF 3D location system is applied to improve the accuracy. RF receivers are preinstalled outside around building and an RF transmitter is attached on a foot. The position of a foot is computed using time-of-arrival from the transmitter to each receiver. In [18], the position error from dead reckoning is corrected by deploying ultrasound beacons as landmarks. Another method is using radar in [19] and distance sensors in [20] to improve the accuracy. Since these papers use a floor as the reference plane, the distance sensor gives information on the foot height. Although there is no installation requirement as the methods in [16,17,18], the methods in [19,20] only improve accuracy of the foot height estimation but do not improve accuracy of horizontal position estimation. Since the foot height is an important information in the gait analysis, the methods in [19,20] can be used effectively for the gait analysis. However, the horizontal position (that is, a person’s position) is a key information in the pedestrian navigation. Thus, the methods in [19,20] are not suitable for pedestrian navigation.

Our method improves the estimation accuracy using a distance sensor in addition to foot-mounted IMUs. We recognize that an important problem in pedestrian navigation is heading correction, and we use a vertical plane (such as a wall) as a reference plane to update heading and position. The proposed system does not require any installation in the environment and any prior knowledge on environment (such as a map).

## 2. Overview System

**Figure 1:**
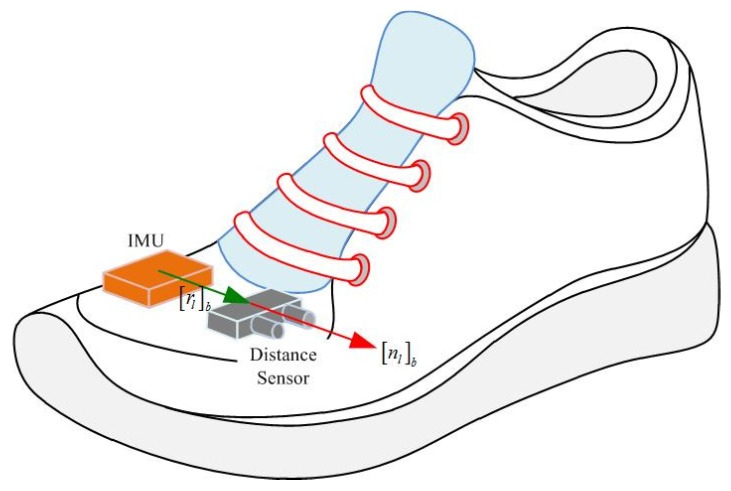
IMU and distance sensor on a shoe.

An IMU (Xsens MTi) is attached on the shoe as in Figure 1. The IMU contains three-axis accelerometers and gyroscopes with 100 Hz sampling frequency. A LIDAR (light detection and ranging, model LL-905-PIN-01 [22]) is also attached on the shoe and used as a distance sensor. The distance is obtained by measuring flight time of infrared light with 30 m measurement range and 33.33 Hz sampling frequency. Body coordinate system (BCS) and world coordinate system (WCS) are used in this paper. The BCS coincides with the IMU coordinate system. The *z* axis of the WCS is pointing upward while the *x* and *y* axes are chosen arbitrarily. The origin of the WCS is assumed to be on the floor. The notation ${\left[a\right]}_{w}$ $\left({\left[a\right]}_{b}\right)$ is used to denote that vector *a* is represented in the world (body) coordinate system.

To compute a LIDAR point’s position, parameters of distance sensor such as the position and pointing direction with respect to BCS are required. In Figure 1, vector ${\left[r_{l}\right]}_{b}\in R^{3}$ denotes the position of a distance sensor while unit vector ${\left[n_{l}\right]}_{b}\in R^{3}$ represents the pointing direction.

The distance sensor parameters (${\left[r_{l}\right]}_{b}$, ${\left[n_{l}\right]}_{b}$ in Figure 1) can be determined using a ruler and a protractor. However, it is not easy to get high accurate parameters using this method. Thus, the parameters are calibrated in the next section.

The main idea of this paper is using vertical planes (such as walls) along a walking path to update position and heading. For example, if a person is walking on a long straight corridor, we can use the wall as a reference to update position and heading. The proposed algorithm automatically detects existence of vertical planes (such as walls) and uses them as references. Thus, no prior map information is required.

**Figure 2:**
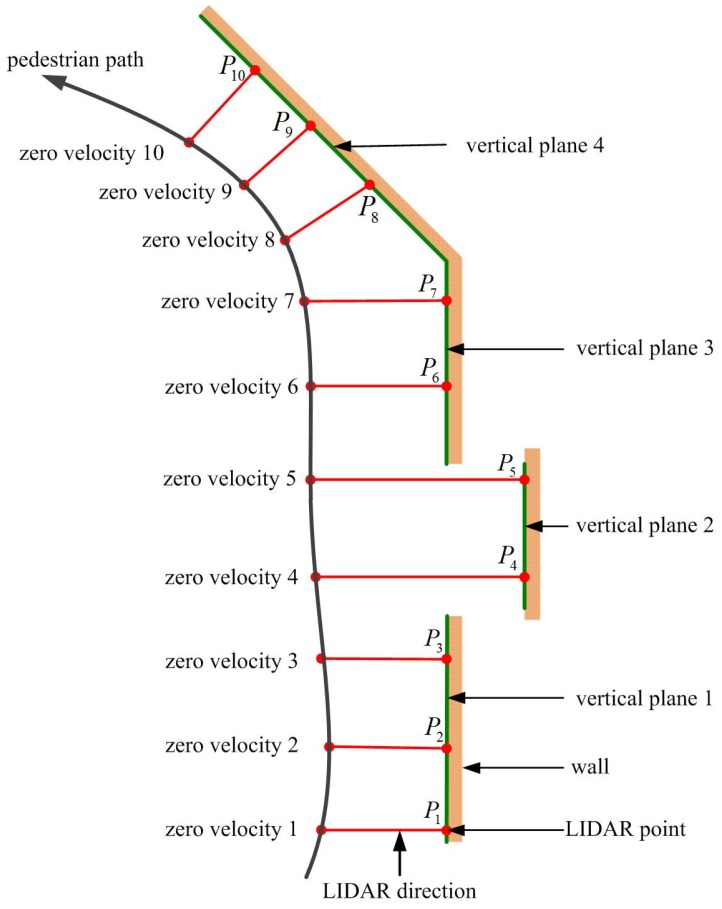
Vertical plane detection using LIDAR.

The detection of vertical planes during walking is explained using an example indoor environment in Figure 2. Suppose a person is walking along the black colored pedestrian path. While walking, a foot touches the floor almost periodically. When a foot is on the floor, the velocity of a foot is zero, and this time interval is called a zero velocity interval (ZVI). At each ZVI *i*, we compute LIDAR point $P_{i}$ based on position and heading estimated using the INA. At ZVI 2, the vertical plane 1 is defined using all LIDAR points between $P_{1}$ and $P_{2}$ (including $P_{1}$ and $P_{2}$). Now, suppose a person is at the ZVI 3 and LIDAR point $P_{3}$ is computed. If $P_{3}$ is close to the vertical plane 1, it is assumed that $P_{3}$ is on the vertical plane 1. Since we know the vertical plane 1 equation and the distance from the vertical plane 1, we can update position and heading. At ZVI 4, LIDAR point $P_{4}$ is not near the vertical plane 1. At this time, vertical plane 1 is not used to update position and heading. At ZVI 5, a new vertical plane is formed. This process is repeated during walking.

## 3. Distance Sensor Calibration

In this section, the sensor unit is handheld while LIDAR is pointing at a floor for the calibration. The coordinate of a LIDAR point with respect to the WCS is computed as follows:

(1) $${\left[b\right]}_{w}=C_{b}^{w}{\left[b\right]}_{b}+r=C_{b}^{w}\left(\left[r_{l}\right]+d{\left[n_{l}\right]}_{b}\right)+r$$

where *r* is the position of IMU with respect to WCS, $C_{b}^{w}$ is the rotation matrix from BCS to WCS, ${\left[b\right]}_{b}=\left[r_{l}\right]+d{\left[n_{l}\right]}_{b}$ is the position of LIDAR point with respect to BCS and *d* is the distance from a distance sensor to LIDAR point. *r* and $C_{b}^{w}$ can be computed from the INA.

**Figure 3:**
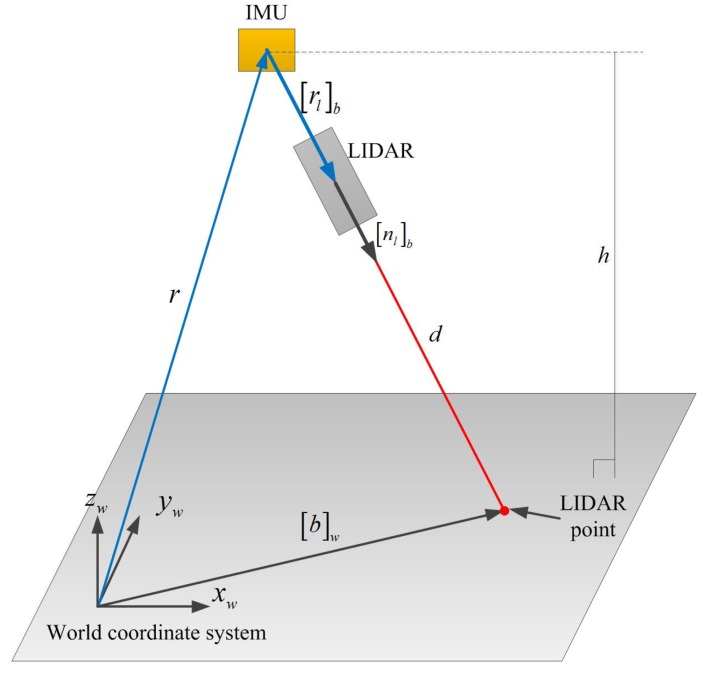
LIDAR calibration (the sensor unit is handheld while LIDAR is pointing at the floor for the calibration).

We setup the distance sensor and IMU so that ${\left[r_{l}\right]}_{b}=k{\left[n_{l}\right]}_{b}$ (see Figure 1). Since ${\left[n_{l}\right]}_{b}$ is a unit vector, *k* is the distance between IMU and the distance sensor. Since *k* ( 3 cm) is very small compared with *d* (1∼5 m), *k* is measured by a ruler. Figure 3 describes our method to estimate ${\left[n_{l}\right]}_{b}$. In the calibration process, LIDAR is pointed at a floor with different poses, where the height is measured with a ruler.

Since ${\left[r_{l}\right]}_{b}=k{\left[n_{l}\right]}_{b}$, Equation (1) becomes

(2) $${\left[b\right]}_{w}=C_{b}^{w}\left(k+d\right){\left[n_{l}\right]}_{b}+r$$

Since the floor is flat and horizontal, the height of IMU is identified by

(3) $$\left[\begin{array}{ccc} 0 & 0 & 1 \end{array}\right]\left(k+d\right)C_{b}^{w}{\left[n_{l}\right]}_{b}=-h$$

where *h* is the height of the IMU, which is measured by a ruler.

If we repeat the measurement *n* times with different poses, we have

(4) $$\left[\begin{array}{c} \left[\begin{array}{ccc} 0 & 0 & 1 \end{array}\right]\left(k+d_{1}\right)C_{1,b}^{w}\\ \left[\begin{array}{ccc} 0 & 0 & 1 \end{array}\right]\left(k+d_{2}\right)C_{2,b}^{w}\\ \vdots \\ \left[\begin{array}{ccc} 0 & 0 & 1 \end{array}\right]\left(k+d_{n}\right)C_{n,b}^{w} \end{array}\right]{\left[n_{l}\right]}_{b}=\left[\begin{array}{c} -h_{1}\\ -h_{2}\\ \vdots \\ -h_{n} \end{array}\right]$$

${\left[n_{l}\right]}_{b}$ is estimated by minimizing the following

(5) $$∥S_{1}{\left[n_{l}\right]}_{b}-S_{2}{∥}_{2}^{2}$$

where

$$S_{1}=\left[\begin{array}{c} \left[\begin{array}{ccc} 0 & 0 & 1 \end{array}\right]\left(k+d_{1}\right)C_{1,b}^{w}\\ \left[\begin{array}{ccc} 0 & 0 & 1 \end{array}\right]\left(k+d_{2}\right)C_{2,b}^{w}\\ \vdots \\ \left[\begin{array}{ccc} 0 & 0 & 1 \end{array}\right]\left(k+d_{n}\right)C_{n,b}^{w} \end{array}\right]\text{ and }S_{2}=\left[\begin{array}{c} -h_{1}\\ -h_{2}\\ \vdots \\ -h_{n} \end{array}\right]$$

The analytic solution to the least squares problem is given by

$${\left[n_{l}\right]}_{b}={\left(S_{1}^{T}S_{1}\right)}^{-1}S_{1}^{T}S_{2}$$

## 4. Kalman Filter Combining an INA and Compensation Using LIDAR

### 4.1. Basic INA

In this subsection, a basic INA is given. This basic algorithm is not a new result and is from [23,24].

Let $v\in R^{3}$ and $r\in R^{3}$ denote the velocity and position of IMU with respect to the WCS. Let $C\left(q\right)\in R^{3\times 3}$ be the direction cosine matrix corresponding to the quaternion [25] $q\in R^{4}$ which represents the rotation relationship between the BCS and WCS.

The quaternion, velocity and position are related as follows [23]:

(6) $$\begin{array}{ccc} \dot{q} & = & \frac{1}{2}\left[\begin{array}{cccc} 0 & -\omega_{x} & -\omega_{y} & -\omega_{z}\\ \omega_{x} & 0 & \omega_{z} & -\omega_{y}\\ \omega_{y} & -\omega_{z} & 0 & \omega_{x}\\ \omega_{z} & \omega_{y} & -\omega_{x} & 0 \end{array}\right]q\\ \dot{v} & = & C{\left(q\right)}^{T}{\left[a\right]}_{b}\\ \dot{r} & = & v \end{array}$$

where *ω* is the angular velocity of the BCS with respect to the WCS and ${\left[a\right]}_{b}\in R^{3}$ is the acceleration in the BCS.

The gyroscope output ($y_{g}\in R^{3}$) and accelerometer output ($y_{a}\in R^{3}$) are given by

(7) $$\begin{array}{ccc} y_{g} & = & \omega +v_{g}+b_{g}\\ y_{a} & = & {\left[a\right]}_{b}+C\left(q\right){\left[\tilde{g}\right]}_{w}+v_{a}+b_{a} \end{array}$$

where ${\left[\tilde{g}\right]}_{w}\in R^{3}$ is the local gravitational vector in the WCS. $b_{g}\in R^{3}$ and $b_{a}\in R^{3}$ are biases of gyroscope and accelerometer, respectively.

The numerical integration algorithm to integrate Equation (6) (replacing ${\left[a\right]}_{b}$ by $y_{a}-C\left(q\right)\tilde{g}$ and replacing *ω* by $y_{g}$) is given in [26]. Let $\hat{q}$, $\hat{r}$ and $\hat{v}$ be the integrated values.

There are errors in $\hat{q}$, $\hat{r}$ and $\hat{v}$ due to sensor noises, represented by $\bar{q}\in R^{3}$, $\bar{r}\in R^{3}$ and $\bar{v}\in R^{3}$:

(8) $$\begin{array}{ccc} \bar{q} & = & \left[\begin{array}{cc} 0_{3\times 1} & I_{3} \end{array}\right]\left({\hat{q}}^{*}⊗q\right)\\ \bar{r} & = & r-\hat{r}\\ \bar{v} & = & v-\hat{v} \end{array}$$

where ⊗ denotes the quaternion multiplication and $q^{*}$ is the conjugate quaternion of *q*. The three dimensional (instead of four dimensional) error description of the quaternion in Equation (8) is from [27].

The state of a Kalman filter is defined by

(9) $$x=\left[\begin{array}{c} \bar{q}\\ b_{g}\\ \bar{r}\\ \bar{v}\\ b_{a} \end{array}\right]\in R^{15\times 1}$$

The system equation for the Kalman filter is given by [28]:

(10) $$\dot{x}\left(t\right)=A\left(t\right)x\left(t\right)+w\left(t\right)$$

where

$$A\left(t\right)=\left[\begin{array}{ccccc} \left[-y_{g}\times \right] & -\frac{1}{2}I & 0 & 0 & 0\\ 0 & 0 & 0 & 0 & 0\\ 0 & 0 & 0 & I & 0\\ -2C{\left(\hat{q}\right)}^{T}\left[y_{a}\times \right] & 0 & 0 & 0 & 0\\ 0 & 0 & 0 & 0 & 0 \end{array}\right],\;\;w\left(t\right)=\left[\begin{array}{c} -\frac{1}{2}v_{g}\\ {w_{b}}_{g}\\ 0\\ -C{\left(\hat{q}\right)}^{T}v_{a}\\ {w_{b}}_{a} \end{array}\right]$$

$\left[a\times \right]\in R^{3\times 3}$ is a skew symmetric matrix corresponding to a vector $a\in R^{3\times 1}$. The noises $w_{bg}$ and $w_{ba}$ represent small variation of biases.

Two measurement equations are used in this paper. The first one is the measurement equation based on the ZVIs, and the other is the measurement equation using the distance sensor (which is given in Section 4.2).

When a foot is touching the ground during walking, its velocity must be zero. This leads to resetting the velocity error in INA. In [29], ZVIs are detected directly using a Doppler velocity sensor. However, ZVIs can be detected indirectly using zero velocity detection algorithms [30,31].

A simple zero velocity detection algorithm is used in this paper. If the following conditions are satisfied, the discrete time index *k* is assumed to belong to ZVIs

(11) $$\begin{array}{cc} ∥y_{g,i}∥\leq B_{g}, & k-\frac{N_{g}}{2}\leq i\leq k+\frac{N_{g}}{2}\\ ∥y_{a,i}-y_{a_{i-1}}∥\leq B_{a}, & k-\frac{N_{a}}{2}\leq i\leq k+\frac{N_{a}}{2} \end{array}$$

where $N_{g}$ and $N_{a}$ are integers.

During ZVIs, we have the following zero velocity updating equation:

(12) $$z_{v}=H_{v}x+v_{zero}$$

where

$$z_{v}=0_{3\times 1}-\hat{v}\in R^{3\times 1}$$

$$H_{v}=\left[\begin{array}{ccc} 0_{3\times 9} & I_{3} & 0_{3\times 3} \end{array}\right]$$

### 4.2. Proposed Position and Heading Updating Algorithm Using the Distance Sensor

**Figure 4:**
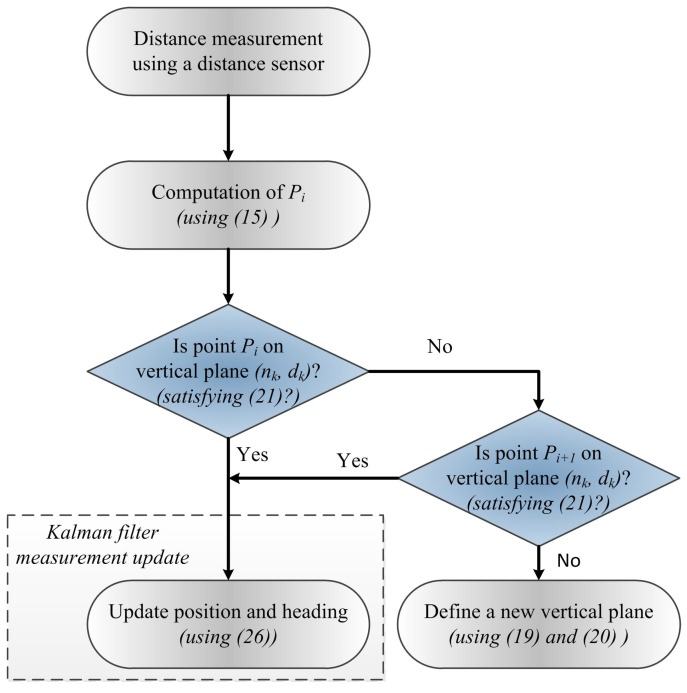
Proposed position and heading updating algorithm using the distance sensor.

The proposed position and heading updating algorithm is shown in Figure 4. As illustrated in Figure 2, the basic idea of the proposed algorithm is to use vertical planes as references to update position and heading. The key issue is when to form a vertical plane and when to abandon an existing vertical plane. This issue is first discussed. At ZVI *i*, LIDAR point $P_{i}$ is given by

(13) $${\left[P_{i}\right]}_{w}=C_{i,b}^{w}{\left[b_{i}\right]}_{b}+r_{i}$$

where $C_{i,b}^{w}\in R^{3\times 3}$ is a rotation matrix from BCS to WCS, and ${\left[b_{i}\right]}_{b}\in R^{3}$ is a LIDAR point with respect to BCS at ZVI *i*.

If there is no defined vertical plane, all LIDAR points in one walking step which is identified by ZVI i−1 and *i* (i>1) are used to define the first vertical plane. All of these points could be on a vertical plane (such as a wall). On the other hand, it is also possible that these points are not on a plane. For example, if there is a passing person, some LIDAR points could be from the person. In addition, it is possible that some points are from a door, which is not on the wall. We first make an assumption that all LIDAR points are on the same plane and derive the plane equation. After that, we verify the same plane assumption in Equation (18). Assume that $P_{i,j}\in R^{3\times 1}\left(1\leq j\leq N\right)$ are all LIDAR points during the step *i*-th (*N* is number of LIDAR points in the step), and $\left(n_{i},d_{i}\right)$ are the vertical plane equation parameters satisfying

(14) $$n_{i}^{T}P_{i,j}=d_{i}$$

The plane equation parameters $\left(n_{i},d_{i}\right)$ are computed by minimizing the following:

(15) $$∥S_{3}\frac{n_{i}}{d_{i}}-S_{4}{∥}_{2}^{2}$$

where

$$S_{3}=\left[\begin{array}{c} P_{i,1}^{T}\\ P_{i,2}^{T}\\ \cdots \\ P_{i,N}^{T} \end{array}\right]\text{ and }S_{4}=\left[\begin{array}{c} 1\\ 1\\ \cdots \\ 1 \end{array}\right]$$

The minimizing solution is given by

(16) $$\frac{n_{i}}{d_{i}}={\left(S_{3}^{T}S_{3}\right)}^{-1}S_{3}^{T}S_{4}$$

Since $n_{i}$ is a normal vector, we have

(17) $$\begin{array}{c} d_{i}=\frac{1}{∥{\left(S_{3}^{T}S_{3}\right)}^{-1}S_{3}^{T}S_{4}∥}\\ n_{i}=d_{i}{\left(S_{3}^{T}S_{3}\right)}^{-1}S_{3}^{T}S_{4} \end{array}$$

Now, we check whether the LIDAR points are from the same vertical plane. If LIDAR points are near the plane $\left(n_{i},d_{i}\right)$ (first and second conditions in Equation (18)) and the plane is vertical (third condition in Equation (18)), we assume that LIDAR points are from the same vertical plane.

(18) $$\;\left\{\begin{array}{c} \frac{1}{N}\sum_{j=1}^{N}\left|n_{i}^{T}P_{i,j}-d_{i}\right|\leq \alpha_{mean}\\ \max\left(|n_{i}^{T}P_{i,j}-d_{i}|\right)\leq \alpha_{max}\\ |\cos^{-1}\left(n_{i}\left(3\right)\right)-90^{0}|\leq \alpha_{angle} \end{array}\right.$$

where $\alpha_{mean}$, $\alpha_{max}$ and $\alpha_{angle}$ are threshold parameters. Note that the inclination angle of the plane equation $\left(n_{i},d_{i}\right)$ is given by $\cos^{-1}\left(n_{i}\left(3\right)\right)$. If Equation (18) is satisfied, the estimated plane $\left(n_{i},d_{i}\right)$ becomes the first defined vertical plane. If Equation (18) is not satisfied, the first vertical plane is searched in the next step.

Now, we have the vertical plane equation $\left(n_{k},d_{k}\right)$ and the next LIDAR point $P_{i}$ is obtained. If this point belongs to the vertical plane, we use the plane equation to update position and heading of a pedestrian. The LIDAR point $P_{i}$ is determined to belong to the vertical plane $\left(n_{k},d_{k}\right)$ if the following is satisfied

(19) $$|n_{k}^{T}P_{i}-d_{k}|\leq \alpha_{plane}$$

where $\alpha_{plane}$ is threshold.

**Figure 5:**
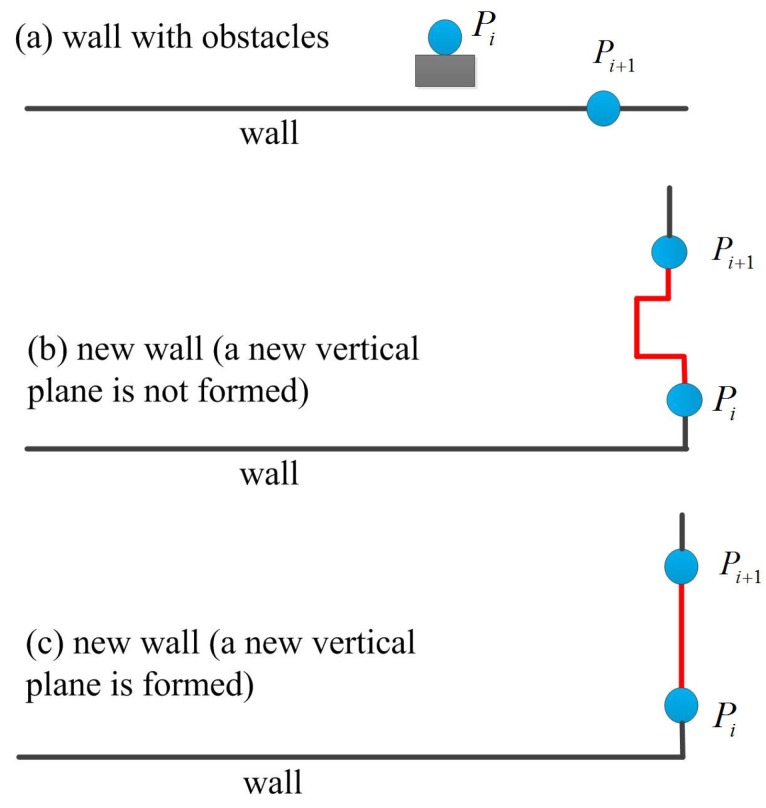
New vertical plane detection.

If LIDAR point $P_{i}$ does not satisfy Equation (19), the point $P_{i}$ could be from an obstacle (such as passing person or door) or could be from a new wall. For example, LIDAR point $P_{i}$ in Figure 5a is from an obstacle (the next LIDAR point $P_{i+1}$ satisfies Equation (19)) while the point $P_{i}$ in Figure 5b,c is from a new wall ( $P_{i+1}$ does not satisfy Equation (19)). In the proposed algorithm, a new vertical plane is only formed using Equation (17) when Equation (18) is satisfied (Figure 5c).

We are going to derive position and heading updating algorithm using LIDAR point $P_{i}$ when the point belongs to the vertical plane $\left(n_{k},d_{k}\right)$. Since $P_{i}$ is on the plane $\left(n_{k},d_{k}\right)$, we have (see Equation (13))

(20) $$n_{k}^{T}\left(C^{T}{\left[b_{i}\right]}_{b}+r_{i}\right)=d_{k}$$

where *C* denotes $C_{w}^{b}\left(q_{i}\right)$. From [32], we have

(21) $$C{\left(q\right)}^{T}=C{\left(\hat{q}\right)}^{T}+2C{\left(\hat{q}\right)}^{T}\left[\bar{q}\times \right]$$

From Equation (20), we have

(22) $$n_{k}^{T}\left(\left(C{\left({\hat{q}}_{i}\right)}^{T}+2C{\left({\hat{q}}_{i}\right)}^{T}\left[{\bar{q}}_{i}\times \right]\right){\left[b_{i}\right]}_{b}+{\hat{r}}_{i}+{\bar{r}}_{i}\right)-d_{k}=0$$

Equation (22) can be rewritten as follows:

(23) $$n_{k}^{T}\left(C{\left({\hat{q}}_{i}\right)}^{T}{\left[b_{i}\right]}_{b}+{\hat{r}}_{i}\right)-d_{k}=2n_{k}^{T}C{\left({\hat{q}}_{i}\right)}^{T}\left[{\left[b_{i}\right]}_{b}\times \right]{\bar{q}}_{i}-n_{k}^{T}{\bar{r}}_{i}$$

This equation is used as a measurement equation in Kalman filter when $P_{i}$ belongs to the vertical plane

(24) $$z_{p}=H_{p}x+v_{p}$$

where $v_{p}$ is a measurement noise and

$$z_{p}=n_{k}^{T}\left(C{\left({\hat{q}}_{i}\right)}^{T}{\left[b_{i}\right]}_{b}+{\hat{r}}_{i}\right)-d_{k}\;H_{p}=\left[\begin{array}{ccccc} 2n_{k}^{T}C{\left({\hat{q}}_{i}\right)}^{T}\left[{\left[b_{i}\right]}_{b}\times \right] & 0_{1\times 3} & -n_{k}^{T} & 0_{1\times 3} & 0_{1\times 3} \end{array}\right]$$

## 5. Experiments and Results

**Figure 6:**
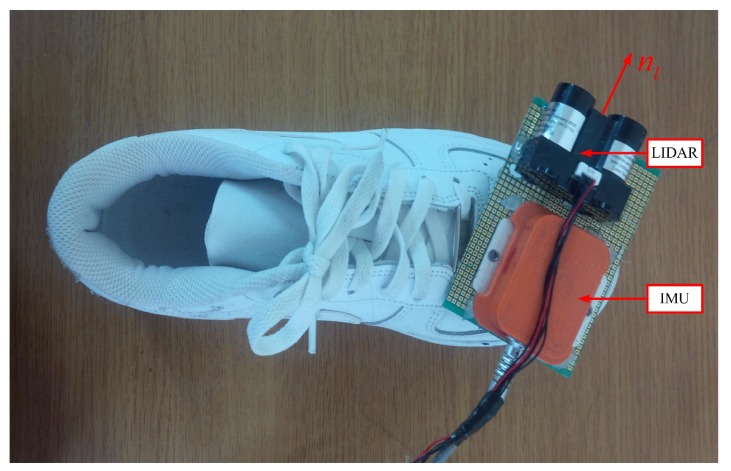
IMU and LIDAR system for the experiment.

Three experiments are performed to verify the proposed algorithm. In the experiments, an IMU (Xsens MTi sensor unit) and distance sensor (LIDAR) are attached on the shoe as shown in Figure 6. Since the algorithm verification is the main purpose of the experiments, the measurement data are collected and processed in Matlab offline.

**Figure 7:**
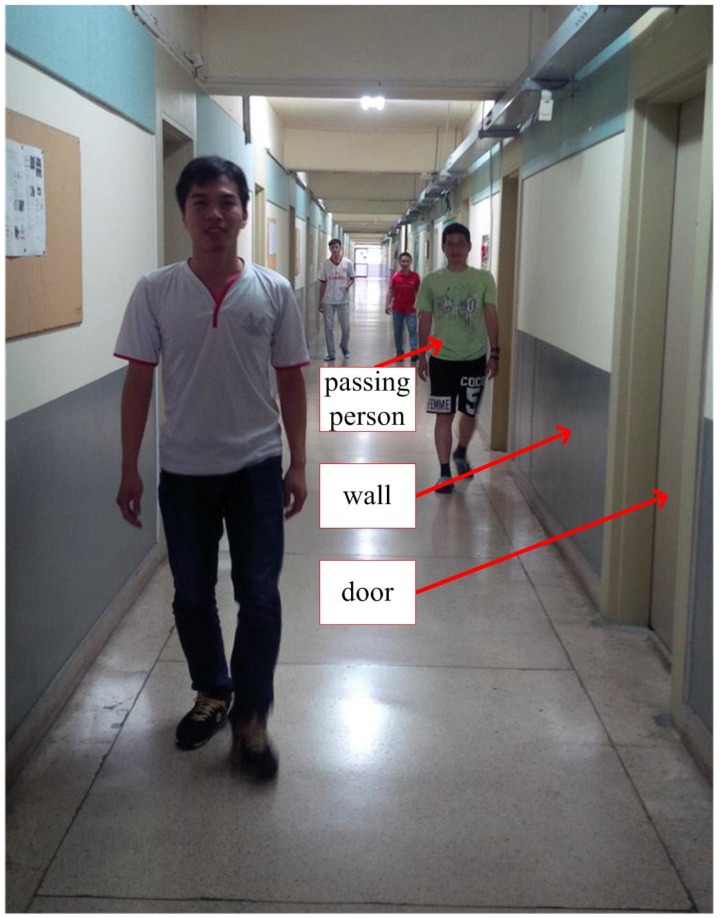
Corridor with doors and passing persons.

**Figure 8:**
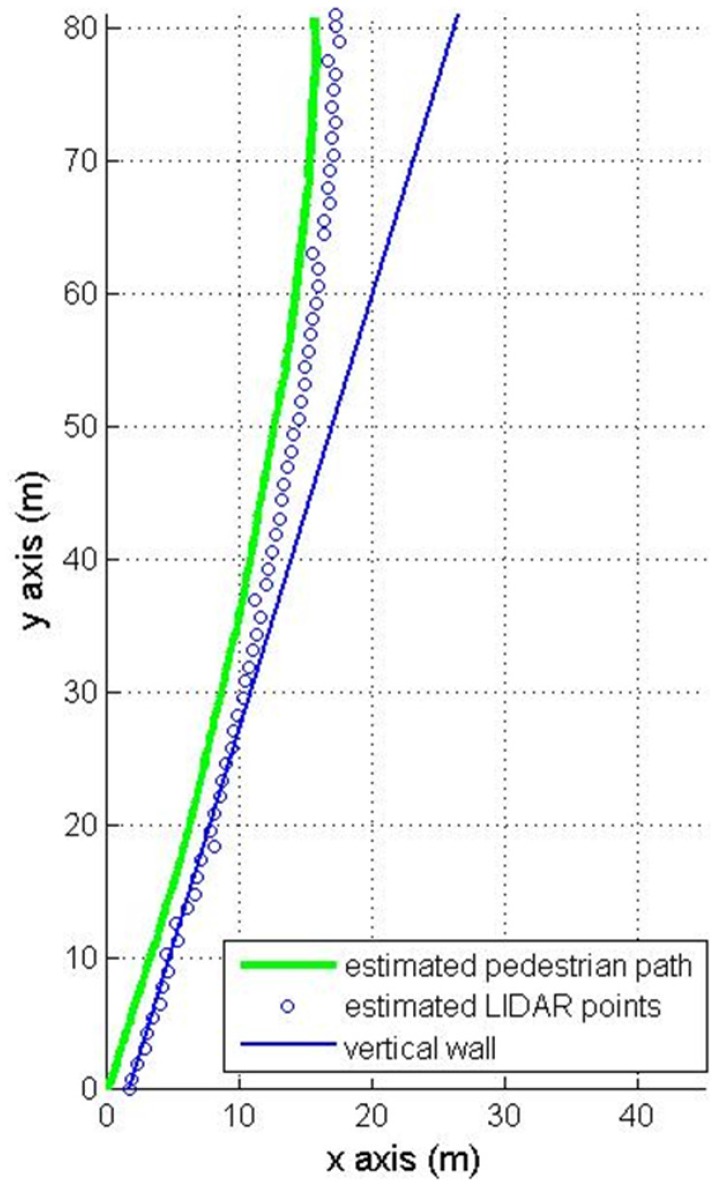
Pedestrian path using a pure INA.

**Figure 9:**
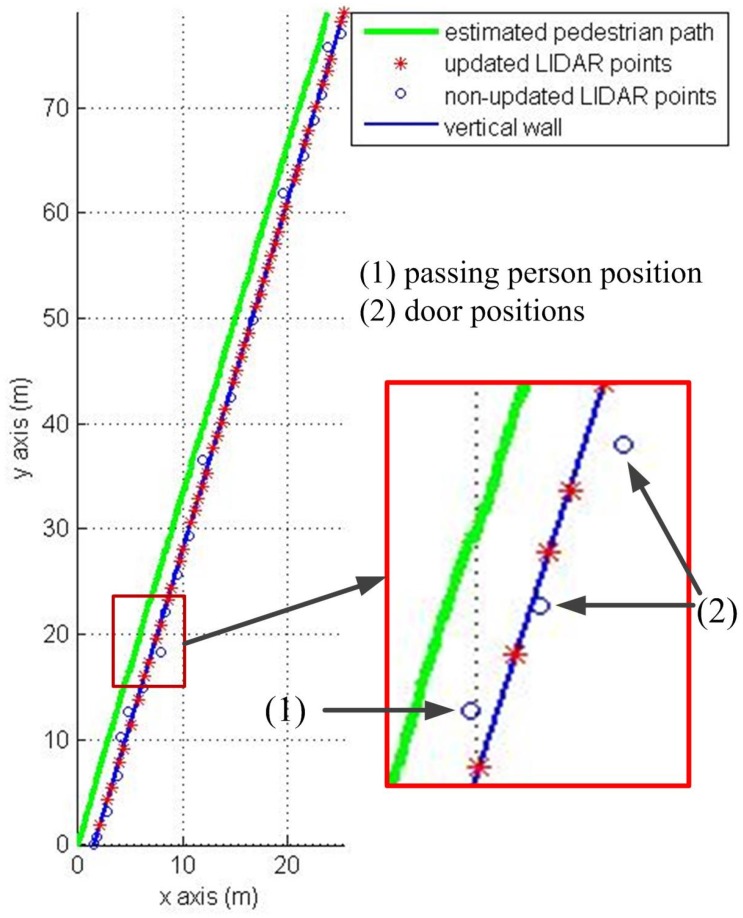
Pedestrian path using the proposed method.

The first experiment is walking along a corridor (84 m) with many doors and passing persons as in Figure 7. The results of the first experiment are shown in Figure 8 (pure INA) and Figure 9 (the proposed algorithm). In the figures, the pedestrian path is drawn in a green color and the vertical wall plane is expressed by the blue line. If LIDAR points satisfy Equation (19), they are used for the measurement update in Equation (24). These “updated LIDAR points” are represented by red “*” symbols. Those points not used for the measurement update are represented by blue “o” symbols. In Figure 8, the estimated pedestrian path tends to drift over time since there is no updating in theheading. In Figure 9, the estimated pedestrian walking path is more accurate since the heading is corrected using the wall information. As can be seen in the zoomed area in Figure 9, the proposed algorithm is working robustly even with many doors and passing persons.

The final position errors after 84 m walking (Figure 8 and Figure 9) are given in Table 1. The root mean square (RMS) position error of a pure INA is 9.23 m. The proposed method gives a better RMS error 1.54 m.

**Table 1:** Final position error comparison in the straight line corridor walking (unit: m).

Experiment Number	Position Error (Pure INA)	Position Error (Proposed Method)
1	4.38	1.74
2	9.48	1.55
3	10.48	1.54
4	11.61	1.40
5	8.50	1.43
**RMS**	**9.23**	**1.54**

Walking is a complex process which depends a lot on each person’s walking style. To see whether the proposed method is affected by different walking styles, the proposed method is tested with five subjects. Each subject is asked to walk 30 m along a corridor three times (thus there are total 15 walking), and the result is shown in Table 2. As can be seen from Table 2, the proposed algorithm can work well independent of subjects, and the position error mean is significantly reduced from 2.03 m (using pure INA) to 0.42 m (using the proposed method).

**Figure 10:**
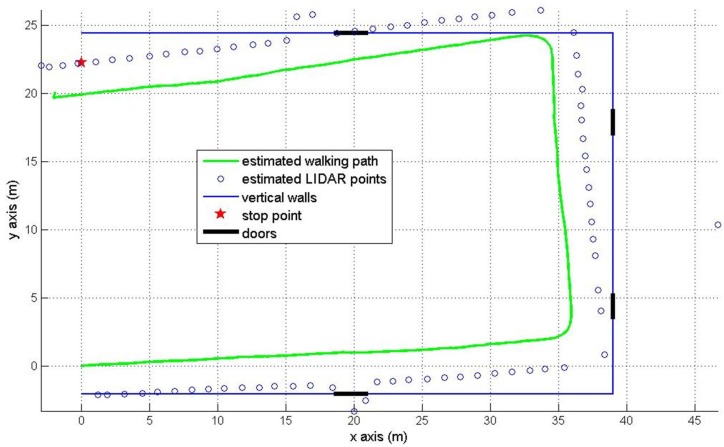
Pedestrian path in the U shaped corridor using a pure INA.

**Figure 11:**
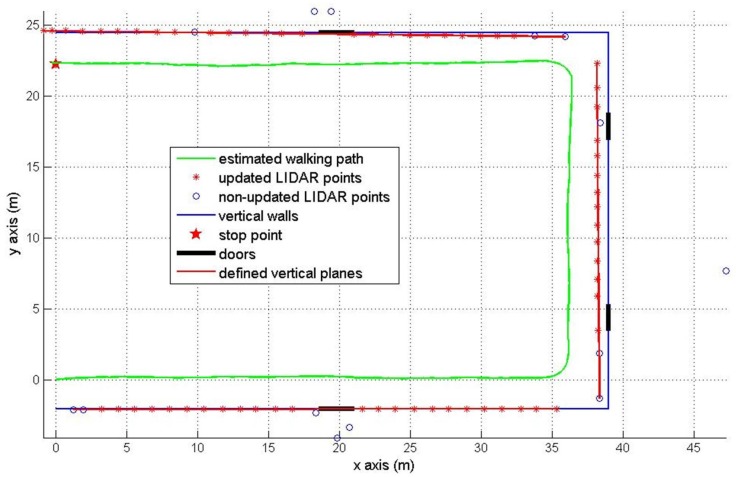
Pedestrian path in the U shaped corridor using the proposed method.

The second experiment is about walking around a U shaped (39×26.5 m) corridor with starting position (0, 0) and final position (0, 22.26 m). The final position is marked by the red star in Figure 10 and Figure 11. There are four doors which are colored in black. The estimated pedestrian path are shown in Figure 10 (pure INA) and Figure 11 (the proposed method). In Figure 10, the heading tends to drift over time since the heading is not corrected as in the Figure 8 case. In Figure 11, we can notice that the heading is effectively corrected using the wall information.

The RMS position error of a pure INA is 3.88 m. The proposed method gives a better RMS error 0.58 m using the wall information (see Table 3).

**Figure 12:**
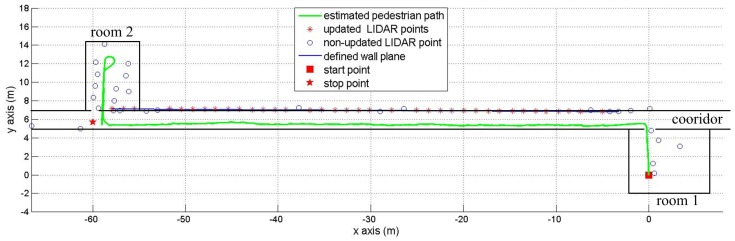
Pedestrian with complex path.

The third experiment is to show that our method can work well with a complex map. In Figure 12, a person started from room 1 and arrived at room 2 after walking a 60 m corridor. Finally, the person went around room 2 and came back to the corridor. The red square in Figure 12 is the starting position, the estimated pedestrian path is shown in the green color and the red star is the final position. We can see that the vertical plane updating is not used inside rooms 1 and 2 (there is no updated LIDAR point inside rooms) due to obstacles.

**Table 2:** Final position error comparison in the corridor walking with different walking style.

Experiment Number	Walking Speed (km/h)	Stride Length (m)	Stride Speed (stride/s)	Position Error (Pure INA) (m)	Position Error (Proposed Method) (m)
1	4.14	1.25	0.92	2.04	0.45
2	4.15	1.25	0.92	1.32	0.34
3	4.57	1.25	1.02	2.57	0.52
4	4.59	1.30	0.98	3.21	0.54
5	4.72	1.30	1.01	3.11	0.37
6	5.19	1.50	0.96	2.25	0.43
7	5.43	1.50	1.01	1.67	0.35
8	5.44	1.50	1.01	1.70	0.44
9	5.46	1.50	1.01	2.53	0.52
10	5.53	1.50	1.02	1.22	0.36
11	5.54	1.50	1.03	1.26	0.42
12	5.57	1.50	1.03	2.33	0.37
13	6.06	1.50	1.12	2.58	0.39
14	6.07	1.50	1.12	0.84	0.31
15	6.10	1.50	1.13	1.80	0.51
**Mean**				**2.03**	**0.42**

**Table 3:** Final position error comparison in the U shaped corridor walking (unit: m).

Experiment Number	Position Error (Pure INA)	Position Error (Proposed Method)
1	3.01	0.20
2	5.20	0.64
3	1.94	0.64
4	2.67	0.91
5	5.32	0.13
**RMS**	**3.88**	**0.58**

## 6. Discussion

The experiment in Table 3 demonstrates that the proposed algorithm can work well with a complex walking path. In Figure 12, there are some updated LIDAR points (red “*” symbols) along the corridor, but there is no updated LIDAR point in room 1 and room 2. This means a pure INA is applied in room 1 and room 2 where there are many obstacles and short walls. The vertical wall plane is automatically detected when the pedestrian enters the corridor. Thus, the estimated pedestrian walking path is more accurate while walking along corridors.

Although Table 2 shows that the proposed method can work well with different walking styles, the proposed algorithm could be affected by walking styles since the sensor is mounted on a shoe. The walking style mainly affects the zero velocity detection used in the INA. This paper uses a simple zero velocity algorithm (see Equation (11)) where its parameters ($N_{g}$, $N_{a}$, $N_{g}$ and $N_{a}$) are chosen so that the algorithm can detect ZVIs for most normal walking styles. These fixed parameters are used during all experiments. To show the effect of this algorithm, another experiment is done for a subject who was asked to walk 3.6 m in a straight line with different speeds. An optical marker and an IMU were attached to his shoe. The IMU was used to detect zero velocity points using zero velocity algorithm, and the marker is used to track the trajectory of the foot using a ground truth system (Flex-13 OptiTrack system).

**Figure 13:**
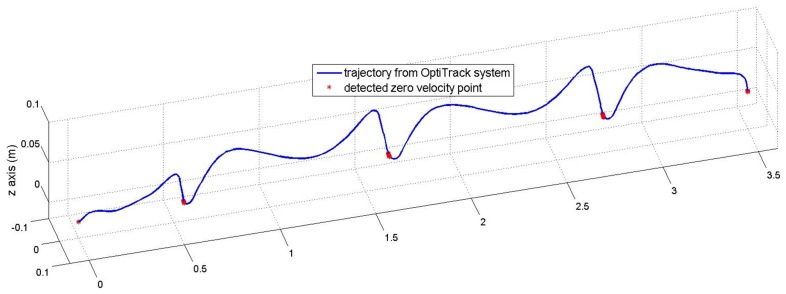
Trajectory of foot and detected zero velocity points.

**Figure 14:**
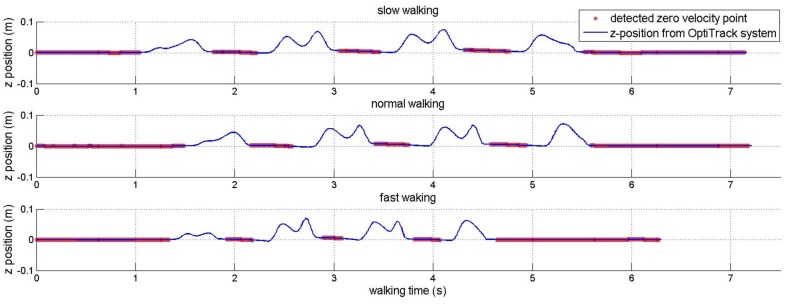
*z*-position of foot and detected zero velocity points.

In Figure 13 and Figure 14, the detected zero velocity points are represented in red “*” symbols and the trajectory of a foot (by an optical tracker) is presented by the blue line. The algorithm can detect ZVIs for three different speeds.

Although the algorithm can detect all occurences of ZVIs, the detected length of ZVI is shorter than the true length of ZVI due to fixed parameters for all walking speeds. This is the drawback of the simple zero velocity algorithm.

The experiment data in Table 2 are used to verify the robustness of zero velocity detection and the result is shown in Table 4. As can be seen in Table 4, there is no missing ZVI detection with walking speed up to 6 km/h. Thus, our algorithm can work well with different walking styles using the fixed parameters. However, the last seven pieces of data, which were obtained by asking subjects to walk as fast as possible, show the drawback of this algorithm. The algorithm with fixed parameters can not detect ZVIs in very high speed walking. For example, there are 22 ZVIs, but the zero velocity algorithm only successfully detects four of them in the last data.

The drawback can be solved by using adjustable parameters for zero velocity detection. For example, the parameters will be adjusted based on the current velocity of the foot. The drawback also can be solved by directly measuring ground touching intervals (for example, using force sensors).

**Table 4:** Zero velocity detection with different walking styles.

Stride Speed (Stride/s)	Walking Speed (km/h)	Stride Length (m)	Number Zero Velocity Interval (True)	Missing Zero Velocity Detection
0.92	4.14	1.25	25	0
0.96	5.19	1.50	21	0
0.98	4.59	1.30	24	0
1.01	4.72	1.30	24	0
1.01	5.46	1.50	21	0
1.01	5.44	1.50	21	0
1.01	5.43	1.50	21	0
1.02	4.57	1.25	25	0
1.02	5.53	1.50	21	0
1.03	5.57	1.50	21	0
1.03	5.54	1.50	21	0
1.12	6.06	1.50	21	0
1.12	6.07	1.50	21	0
1.13	6.10	1.50	21	1
1.16	5.96	1.43	22	4
1.17	6.04	1.43	22	8
1.26	6.78	1.50	21	17
1.28	6.94	1.50	21	18
1.32	6.81	1.43	22	19
1.35	6.95	1.43	22	18

## 7. Conclusions

The proposed method uses vertical planes (such as walls) to improve heading and position estimation accuracy. The vertical planes are constructed using a distance sensor.

The proposed method is verified through three experiments in a straight corridor, a U shaped corridor and a complex walking path. Experiment results in Table 1, Table 2 and Table 3 show that the proposed method gives a better RMS error compared with a pure INA. Furthermore, the zoomed area in Figure 9 indicates that the proposed algorithm is working robustly even with many doors and passing persons.

The paper also shows the drawbacks and solutions of using a simple zero velocity algorithm through the experiment in discussion section.

Since there are many vertical walls indoors, the proposed algorithm can be used for almost any indoor environments. Furthermore, the proposed system does not require any installation on environment and any prior knowledge on environment.

As a future research topic, this paper can be improved by using two distance sensors which point to different sides (left and right). By using two distance sensors, vertical planes (such as walls) in two sides of corridor will be recognized. This could improve our algorithm, especially when one side loses tracking vertical plane due to obstacles or no wall.
